# Development of a Screen-Printable Liquid Metal Ink on PDMS Substrates Toward Flexible Conductive Electronics

**DOI:** 10.3390/s26113279

**Published:** 2026-05-22

**Authors:** Mengwen Guo, Shengming Jin, Sanhu Liu, Fang Wang

**Affiliations:** 1 School of Minerals Processing and Bioengineering, Central South University, Changsha 410083, China; 18954473102@163.com; 2 Key Laboratory for Mineral Materials and Application of Hunan Province, Central South University, Changsha 410083, China; 3 Changsha Advanced Materials Industrial Research Institute Co., Ltd., Changsha 410083, China; liulakes@163.com (S.L.); htxcl@htxcly.cn (F.W.)

**Keywords:** liquid metal ink, screen printing, multi-solvent regulation, PDMS, flexible conductive electronics

## Abstract

In this study, poly(vinylpyrrolidone) (PVP)-modified liquid metal (LM) particles were formulated into a mixed-solvent system comprising ethanol (EtOH), 1,2-propanediol (1,2-PG), and a trace amount of N,N-dimethylformamide (DMF). This design addresses the instability, poor wetting/adhesion on polydimethylsiloxane (PDMS), and limited rheological tunability of conventional LM inks for screen printing. By regulating solvent evaporation during drying, the system enables coordinated control over wettability, viscosity, shear-thinning behavior, and drying-induced film formation. At an LM:PVP weight ratio of 20:1, the contact angle on PDMS decreased from 115° to 17.8°. The ink exhibited pronounced shear-thinning characteristics with tunable viscosity in the range of 1000–3000 cP, meeting the screen-printing requirements of facile mesh passage and rapid setting. Following laser activation, the printed conductive patterns demonstrated stable electrical performance, with a resistance drift of less than 1% after 14 days of storage and a ΔR/R_0_ of less than 1% after 3000 bending cycles at a bending diameter of 1 cm. Furthermore, a resistance drift of less than 3% was observed after 1000 stretching cycles at 30% strain. This study proposes a viable materials and processing strategy for the reliable screen printing of LM:PVP ink on PDMS substrates toward flexible conductive electronics. The motion-monitoring test is presented only as a preliminary proof-of-concept demonstration of motion-induced electrical resistance response, rather than as a sensor performance evaluation.

## 1. Introduction

The rapid advancement of flexible conductive electronics, including soft circuits, deformable interconnects, and wearable systems, has driven an increasing demand for conductive materials amenable to large-area, high-throughput, and cost-effective patterning [1,2,3]. Gallium-based liquid metals (LMs), such as eutectic gallium–indium and gallium–indium–tin alloys, are considered promising candidates owing to their unique combination of metallic conductivity and liquid-like deformability [4]. However, the direct utilization of LM remains constrained by its intrinsically high surface tension [5], poor dispersion stability [6], and weak interfacial adhesion to prevalent elastomeric substrates such as polydimethylsiloxane (PDMS) [7,8]. These limitations impede not only precise pattern formation but also structural reliability of conductive features under mechanical deformation and prolonged operation [9,10]. Therefore, transforming LM into printable inks represents a critical step toward its practical and scalable application in flexible conductive electronic manufacturing [1,11,12].

Among various patterning techniques, screen printing is particularly well-suited for large-scale LM patterning owing to its low cost, high efficiency, and broad applicability [13,14]. Screen-printable LM inks demand both high LM loading for conductivity and appropriate rheological, wetting, and film-forming properties. Specifically, suitable viscosity and thixotropy are essential for mesh filling under squeegee shear and shape retention after printing [15]. Adequate substrate wettability is required to achieve well-defined pattern edges and high printing precision [16]. Nevertheless, the inherent characteristics of LM render it challenging to satisfy these coupled requirements simultaneously [17,18]. Specifically, current LM inks for screen printing face several interrelated challenges, including particle aggregation driven by high surface tension, insufficient rheological tunability for mesh transfer and pattern retention, poor wetting and adhesion on elastomeric substrates, and conductivity degradation due to surface oxidation [11,12,13,19,20]. These challenges are especially acute on low-surface-energy PDMS substrates, where inadequate interfacial compatibility can result in discontinuous patterns, edge defects, and unstable electrical performance.

Various strategies have recently been proposed to improve LM ink stability, printability, and interfacial compatibility. For instance, Cui et al. [11] developed an LM-montmorillonite composite ink to mitigate aggregation and enhance wettability. This enabled printing at a resolution of 50 µm on PDMS. However, the limited LM loading compromised the conductivity required for practical applications. Shang et al. [21] reported a PVP-based LM ink using propylene glycol as a green solvent for fully screen-printed stretchable multilayer circuits, demonstrating stable dispersion, high conductivity, and good stretchability. Nevertheless, that work focused primarily on circuit fabrication and water-spray sintering, whereas the coupled regulation of wetting, rheology, drying-induced film formation, and laser activation on low-surface-energy PDMS substrates remains less explored. Ma et al. [22] prepared a high-concentration LM ink using a confinement-gel strategy to improve stability. However, its elevated viscosity limited screen-printing suitability. Other studies, such as those by Lin et al. [23] and Chiu et al. [24], further improved LM loading, stability, and processability through hydrogel or core–shell designs. Collectively, these advances have expanded the processing window of LM inks. Yet, the integrated control of wettability, rheology, drying behavior, and post-printing activation for screen printing on PDMS remains insufficiently addressed. To distinguish this work from existing LM ink systems, Table 1 summarizes recent strategies for printable LM inks.

To address these issues, this work develops a PVP-modified LM ink based on an EtOH/1,2-PG/DMF mixed-solvent system for screen printing on PDMS substrates. The mixed-solvent system is designed to regulate mesh transfer, spreading, solvent evaporation, and film formation during printing and drying. Combined with the interfacial dispersion and stabilizing effects of PVP, this formulation improves the compatibility between LM particles and low-surface-energy PDMS substrates. Notably, the differential solvent volatility is exploited to moderate the drying process and suppress drying-induced nonuniformity, thereby improving film continuity and pattern uniformity. Following laser activation, the printed patterns exhibit conductivity on the order of 10^5^ S/m and maintain stable resistance responses during storage and repeated deformation. These results demonstrate the potential of the printed patterns for PDMS-based flexible conductive electronics. The motion-monitoring experiment is included solely as a preliminary proof-of-concept demonstration of motion-induced resistance response, rather than as a comprehensive sensor performance evaluation.

## 2. Experimental Section

### 2.1. Materials

Liquid metal (LM, purity ≥ 99.9%) was purchased from Sichuan High-Purity Materials Technology Co., Ltd. (Mianyang, China) Ethanol (EtOH, analytical grade, purity ≥ 99.7%), 1,2-propanediol (1,2-PG, analytical grade, purity ≥ 99.0%), and N,N-dimethylformamide (DMF, analytical grade, purity ≥ 99.5%) were obtained from Sinopharm Chemical Reagent Co., Ltd. (Shanghai, China). Polyvinylpyrrolidone (PVP, K30, molecular weight 40,000) was purchased from Shanghai Aladdin Biochemical Technology Co., Ltd. (Shanghai, China) and used as a dispersant. Polydimethylsiloxane (PDMS, SYLGARD 184) was purchased from Dow Corning. (Shanghai, China). PDMS substrate was prepared by mixing the prepolymer and curing agent at a 10:1 mass ratio, followed by curing at 60 °C for 2 h. Deionized water (resistivity ≥ 18.2 MΩ·cm) was produced using a laboratory ultrapure water system and used throughout all experiments.

### 2.2. Preparation of LM Inks

First, ethanol and 1,2-PG were blended at preset volume fractions to obtain the mixed solvent. Then, a 5 mL aliquot of this solvent was mixed with a small amount of PVP under stirring until complete dissolution. Subsequently, 5 g of bulk LM was introduced, and ultrasonic fragmentation was performed using a cell disruptor (JY92-IIN, Ningbo Scientz Biotechnology Co., Ltd., Ningbo, China) for 15 min. Next, 0.2 mL of DMF was added dropwise, followed by magnetic stirring at 300 r/min for 10 min to ensure homogeneity (85-2, Shanghai Qilinbeier Instrument Co., Ltd., Shanghai, China). After stirring, the mixture was allowed to settle at room temperature for 4 h, and the bottom phase was collected as the target LM ink. Based on the ethanol volume fraction, the inks were designated LMI-PVP-E100, LMI-PVP-E90, LMI-PVP-E70, and LMI-PVP-E50.

### 2.3. Screen Printing and Laser Activation

**Figure 1:**
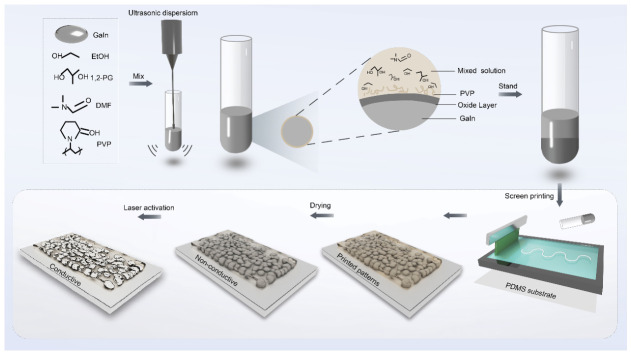
Schematic illustration of LMI-PVP ink preparation and activation. Adapted with modifications from Shang et al. [21] under the terms of the Creative Commons Attribution 4.0 International License.

A PVA release film was first placed on a ceramic plate. The PDMS prepolymer and curing agent were mixed at a mass ratio of 10:1, and the resulting mixture was uniformly coated onto the PVA-covered ceramic plate using an automatic film coater set to a thickness of 1200 μm. The coated film was subsequently cured at 60 °C for 0.5 h. Upon curing, the PDMS film was peeled from the ceramic plate using the PVA film, yielding a flat PDMS substrate for subsequent use. LM inks (LMI-PVP-E90, LMI-PVP-E70, and LMI-PVP-E50) were screen-printed onto PDMS substrates using a manual screen-printing platform (SMT-200, Shenzhen Shiwangda Technology Co., Ltd., Shenzhen, China) equipped with customized stainless-steel screens of 420 and 225 mesh to fabricate LMI-PVP-PDMS samples. The printed patterns were designed in-house. A polyurethane squeegee blade was used at an angle of approximately 45 °C during manual printing, and the distance between the screen and the PDMS substrate was maintained at approximately 0.2 cm to ensure stable ink transfer and printing uniformity. The printing speed was maintained at approximately 50 mm/s. Following printing, the samples were pre-dried at room temperature for 10 min and then thermally cured at 60 °C for 30 min (Figure 1).

Laser activation was performed using a 1064 nm pulsed fiber laser system (Raycus, Wuhan, China, RFL-P50QB, 50 W) equipped with a SINO-GALVO SG7110 galvanometer scanner (SINO-GALVO (Jiangsu) Technology Co., Ltd., Zhenjiang, China). The optimized activation parameters were 10% of the maximum laser power, corresponding to approximately 5 W, and a scanning speed of 1500 mm/s. Upon localized laser irradiation, the PVP dispersant decomposes while the LM particles melt and coalesce into a continuous conductive network, thereby activating the electrical conductivity of the printed ink.

### 2.4. Characterization of LMI-PVP

Scanning electron microscopy (SEM, SU8010, Hitachi High-Technologies Corporation, Tokyo, Japan) was employed to examine particle morphology and aggregation state. At least 200 particles were analyzed per randomly selected field of view using image analysis software (Image-Pro Plus, Media Cybernetics, Rockville, MD, USA) to determine equivalent diameters, average size, and size distribution. Static contact angles on PDMS substrates were measured using a contact angle goniometer (JC2000D, Shanghai Zhongchen Digital Technology Apparatus Co., Ltd., Shanghai, China) to evaluate ink wettability. The viscosity–shear rate relationship was characterized via the cone-and-plate method at 25 °C over a shear rate range of 0.1 to 100 s^−1^ (MCR-302, Anton Paar GmbH, Graz, Austria). Finally, the rheological characteristics of inks with varying solvent ratios and their compatibility with the screen-printing process were analyzed. Printed circuit surface morphology was observed using optical microscopy (OM, VHX-6000, Keyence Corporation, Osaka, Japan), and line width, thickness, and edge definition were measured to evaluate printing resolution across different LMI systems. Adhesion to the PDMS substrate was evaluated using a tape-peeling test (3 M Scotch tape, 3 M Company, St. Paul, MN, USA, 180°peeling), with the residual circuit state examined post-peeling. Post-laser-activation resistance was measured using a resistance tester, and electrical conductivity was calculated from the measured line thickness. In addition, mechanical stability was assessed by measuring resistance after 1000 stretching cycles at 30% strain and 3000 bending cycles (1 cm diameter cylinder). For the storage stability, encapsulated devices were stored flat under ambient air conditions at 25 °C and 60% relative humidity for 14 days. The samples were protected from direct light exposure, and their resistance was measured at the same time each day.

### 2.5. Surface Tension Calculation

The surface tension of the ink was determined from contact angle measurements on PDMS using the Owens–Wendt two-liquid method in combination with Young’s equation [25].

Young’s equation

At the solid–liquid–gas three-phase contact line, interfacial tensions satisfy the force balance condition:

(1) $$\gamma_{SV}=\gamma_{SL}+\gamma_{LV}\times \cos\theta$$

where $\gamma_{SV}$ is the surface tension of the PDMS solid surface; $\gamma_{SL}$ is the solid–liquid interfacial tension between PDMS and the liquid (mN/m); $\gamma_{LV}$ is the liquid surface tension (mN/m); and *θ* is the measured contact angle of the ink on the PDMS surface (°).

Owens–Wendt equation

The solid–liquid interfacial tension can be expressed as the geometric mean of the dispersive and polar components of the surface energy:

(2) $$\gamma_{SL}=\gamma_{SV}+\gamma_{LV}-2\left(\sqrt{\gamma_{SV}^{d}\times \gamma_{LV}^{d}}+\sqrt{\gamma_{SV}^{p}\times \gamma_{LV}^{p}}\right)$$

where $\gamma_{SV}^{d}$ and $\gamma_{SV}^{p}$ represent the dispersive and polar components of the PDMS surface energy (mN/m), respectively; and $\gamma_{LV}^{d}$ and $\gamma_{LV}^{p}$ represent the corresponding components of the liquid surface energy (mN/m).

The total surface tension of the liquid satisfies

(3) $$\gamma_{LV}=\gamma_{LV}^{d}+\gamma_{LV}^{p}$$

Deionized water and α-Bromonaphthalene were employed as probe liquids [26] to determine the surface energy parameters of PDMS via the two-liquid method (Table 2). PDMS molecular chains consist of a Si-O backbone and -CH_3_ side groups. The high proportion of hydrophobic methyl groups yields a surface energy dominated by the dispersive component, while the polar component is considered negligible ($\gamma_{LV}^{p}\approx 0$).

Substituting the probe liquid contact angles into Equations (1)–(3) yields a PDMS surface energy of approximately 23.5 mN/m, consistent with the reported range for pristine PDMS (19–24.1 mN/m) [25,27], thereby validating the rationality of the simplified calculation method. By substituting the measured contact angles into Equations (1)–(3), the surface tension corresponding to each contact angle can be obtained using an iterative method.

## 3. Results and Discussion

### 3.1. PVP Coating Mechanism of LM Particles and Ratio Optimization

The dispersion stability, interfacial compatibility, and conductivity of LM inks are critically governed by particle surface chemistry and the interparticle interactions. During ultrasonic cavitation and fragmentation, an oxide/oxygen-containing layer forms on the LM particle surface, providing active sites for polymer adsorption. PVP anchors to these oxygen-containing species via coordination and hydrogen bonding through its amide carbonyl (C=O) groups, forming a stabilizing interfacial layer. This layer suppresses agglomeration through steric hindrance and modulates particle surface energy, thereby improving colloidal stability, wettability, and spreading behavior.

**Figure 2:**
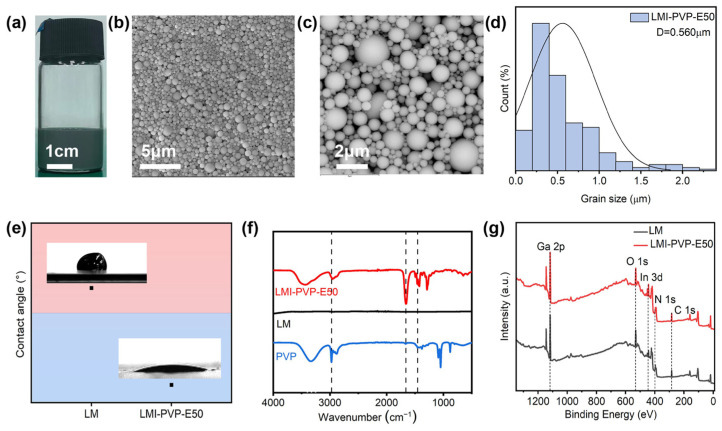
Characterization of LMI-PVP-E50. (**a**) Optical image of LMI-PVP. (**b**,**c**) SEM images of LMI-PVP at different magnifications. Scale bars = 5 μm in (**b**) and 2 μm in (**c**). (**d**) Particle size distribution of LMI-PVP-E50. (**e**) Enlarged contact angle images of LM and LMI-PVP-E50 on PDMS substrates, showing contact angles of 115° and 17.8°, respectively. (**f**) FTIR spectra of LM and LMI-PVP-E50. (**g**) XPS survey spectra of LM and LMI-PVP-E50.

As shown in Figure 2a–d and Figure S1, the PVP-modified LMI-PVP particles exhibit uniform, spherical morphology with an average size of approximately 0.560 µm, and negligible agglomeration. Compared with unmodified LMI, LMI-PVP demonstrates markedly improved dispersion stability, with no significant sedimentation within 48 h and only minor particle growth after 15 days. In contrast, unmodified LMI exhibits distinct stratification after 12 h and severe coarsening after 48 h (Figure S2). As shown in Figure 2e, the contact angle measurements further indicate that PVP modification substantially enhances LM wettability on PDMS, reducing the contact angle to 17.8°. Strong interfacial interaction between PVP and LM particles is corroborated by FT-IR and XPS analyses (Figure 2f,g). Collectively, these results confirm that the PVP coating effectively enhances both dispersion stability and interfacial wettability [28].

**Figure 3:**
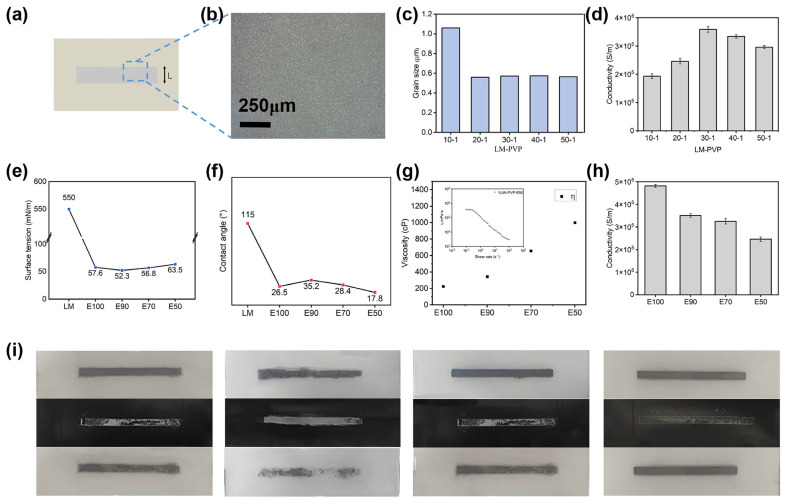
Characterization of liquid metal inks: particle size, wettability, rheology, and electrical performance. (**a**) Schematic diagram of the printed conductive pattern. (**b**) Optical microscopy image of LMI-PVP at an LM:PVP ratio of 20:1. Scale bar = 250 μm. (**c**) Particle size and electrical conductivity of LMI-PVP at LM:PVP ratios of 10:1, 20:1, 30:1, 40:1, and 50:1. (**d**–**g**) Contact angle, surface tension, viscosity (inset: viscosity/shear rate curves of the LMI-PVP-E50 system), and electrical conductivity of LM, LMI-PVP-E100, LMI-PVP-E90, LMI-PVP-E70, and LMI-PVP-E50. (**h**) Electrical conductivity of printed patterns fabricated by LMI-PVP-E100, LMI-PVP-E90, LMI-PVP-E70, LMI-PVP-E50 inks. (**i**) Optical images of printed patterns at V(EtOH):V(1,2-PG) ratios of 50:50 (without DMF), 90:10, 70:30, and 50:50 (**top**: as-printed patterns; **middle**: after tape peeling; **bottom**: comparison of peeled residues).

Varying LM:PVP ratios reveals a trade-off between coating sufficiency and organic phase residue/barrier effects (Figure 3a–d and Figure S3). Excessive PVP (10:1) yields dense surface coating but increases viscosity, suppressing ultrasonic fragmentation and introducing insulating organic residues at the particle interfaces. This limits the formation of conductive pathways. Insufficient PVP (30:1 to 50:1) results in incomplete surface coverage, weakened steric stabilization, and poor tolerance to drying-induced shrinkage, leading to film cracking or rupture. At an LM:PVP ratio of 20:1, these competing effects are balanced, yielding uniform particle dispersion, intact film formation, and a post-activation conductivity of approximately 10^5^ S/m.

### 3.2. Screen-Printable Ink for PDMS Low-Surface-Energy Substrates: Synergistic Optimization of Wettability and Rheology

Based on the optimized LM:PVP ratio of 20:1, the solvent system was further adjusted to balance ink wetting/spreading on low-surface-energy PDMS with screen-printing mesh transfer requirements. A ternary mixed-solvent system comprising EtOH and 1,2-PG as primary solvents and DMF as a co-solvent was designed. Four inks were formulated by varying the EtOH volume fraction from 100% to 50% (denoted LMI-PVP-E100, LMI-PVP-E90, LMI-PVP-E70, and LMI-PVP-E50) to investigate the impact of solvent composition on macroscopic physicochemical properties. As shown in Figure 3e, the contact angle on PDMS decreased from 35.2° to 17.8° with solvent optimization. This indicates improved interfacial compatibility and spreading stability. Note that the surface tension and interfacial energy parameters derived from the contact angle model are used solely to compare interfacial trends rather than to interpret θ independently. Overall, the synergistic regulation of EtOH and 1,2-PG provides a suitable interfacial basis for achieving well-defined pattern edges and stable adhesion during screen printing.

Screen printing requires the ink to flow smoothly through the mesh under shear while retaining pattern integrity upon release, necessitating high viscosity at low shear rates and pronounced shear-thinning behavior at high shear. As shown in the inset of Figure 3g, all inks exhibit obvious shear-thinning behavior over a shear rate range of 0.1 to 100 s^−1^. Decreasing the EtOH content and increasing the 1,2-PG proportion adjusts the viscosity from 100 to 3000 cP. This is attributed to the higher viscosity of 1,2-PG, which increases intermolecular friction and promotes transient weak interactions and chain entanglements between LM particles and PVP chains at low shear rates. As the shear rate increases, these weak structures progressively disintegrate, substantially reducing flow resistance. This rheological profile is favorable for screen printing, enabling efficient mesh transfer under shear and rapid shape retention after printing.

Printed patterns and tape-peeling results (Figure 3i) reveal that the solvent ratio critically affects both printing quality and PDMS adhesion. LMI-PVP-E90 exhibits poor shape retention, insufficient transfer, and limited wettability, resulting in local defects, line discontinuities, and blurred edges. Increasing 1,2-PG content enhances shear-thinning behavior, viscosity, and wettability (lower contact angle). These properties improve line uniformity, film continuity, and interfacial adhesion [29]. Among all formulations, LMI-PVP-E50 delivers optimal performance, producing high-precision patterns with uniform, continuous films free of pinholes and coffee-ring defects. To elucidate the origin of this improved film quality, the drying behavior under multi-solvent regulation was subsequently investigated.

### 3.3. Mechanism of Uniform Drying of LMI-PVP Ink Regulated with a Multi-Solvent System and the Principle of Laser Activation

**Figure 4:**
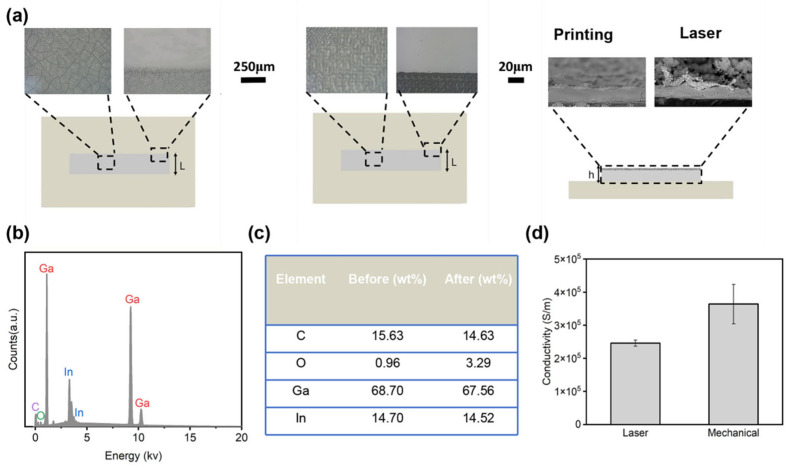
Morphology, composition, and conductivity of the printed conductive patterns. (**a**) Optical microscopy images of different regions of LMI-PVP-E100 and LMI-PVP-E50 after printing, and cross-sectional SEM images of the printed patterns before and after laser activation. (**b**) EDS spectrum of the conductive pattern after laser activation. (**c**) Elemental composition comparison before and after laser activation. (**d**) Electrical conductivity of conductive patterns after laser and mechanical activation. Scale bars = 250 μm in the optical microscopy images and 20 μm in the cross-sectional SEM images.

Post-printing ink drying and film formation are governed by solvent evaporation and particle migration. For the single low-boiling solvent system (LMI-PVP-E100), the evaporation rate is high. Rapid solvent loss induces local solutes/particles enrichment, elevating viscosity and promoting contact line pinning [30]. Additionally, line edges, pore boundaries, and deposit boundaries formed after mesh release serve as high evaporative flux contact line regions. This induces outward compensatory flow and drives particle accumulation at the periphery. Consequently, pronounced coffee-ring deposition, pore formation and nonuniform film thickness (Figure 4a) [31]. In contrast, the mixed-solvent system leverages the pronounced boiling point differences among EtOH (Tb = 78.3 °C), DMF (Tb = 153 °C), and 1,2-PG (Tb = 188.2 °C) to achieve a broader boiling range and slower evaporation. As a result, evaporation-induced internal convection and edge enrichment are effectively suppressed. This promotes uniform particle deposition and dense, continuous film formation with fewer defects. Among these formulations, LMI-PVP-E50 strikes an optimal balance between rapid setting and sufficient leveling, resulting in a more compact and uniform film structure. This also enables a screen-printing resolution of up to 50 µm (Figure S4).

Laser activation converts insulating patterns into conductive networks by disrupting the Ga–O oxide layer on LM particle surface and promoting particle coalescence. EDS analysis (Figure 4b,c and Figure S5) confirms that oxygen content decreases from 3.29% to 0.96% after activation. The uniform film structure achieved through multi-solvent regulation ensures homogeneous laser energy distribution. This helps avoid local overheating in thick regions and insufficient activation in thin regions. Consequently, both laser- and mechanically activated samples exhibit conductivities on the order of 10^5^ S/m (Figure 4d). Although laser activation yields slightly lower conductivity than mechanical activation, it offers superior stability and a narrower process window owing to its higher precision and controllability. This makes laser activation more suitable for the batch fabrication of high-precision, flexible electronics.

### 3.4. Fabrication and Electrical Stability of LMI-PVP-PDMS Flexible Conductive Devices

**Figure 5:**
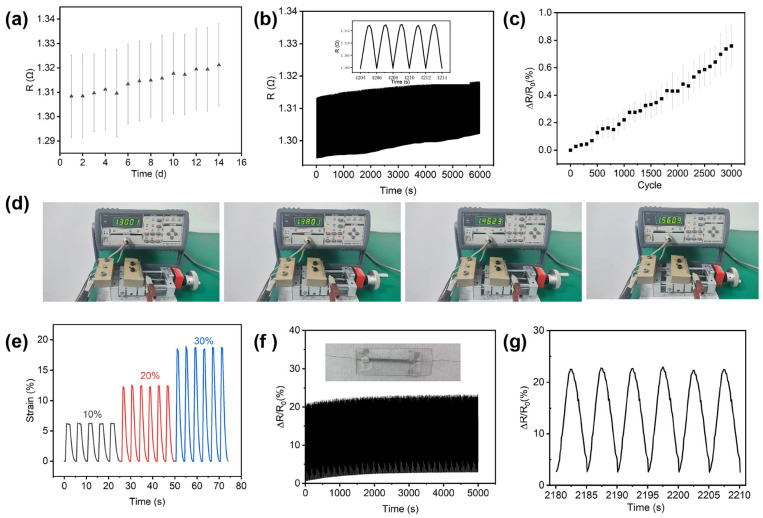
Electrical stability of LMI-PVP-PDMS conductive devices under storage and cyclic deformation. (**a**) Resistance variation in the devices over 14 days of static storage. (**b**,**c**) Resistance change and relative resistance change (ΔR/R_0_) of the devices during 3000 bending cycles. (**d**) Photographs of the devices under 0, 10%, 20%, and 30% strain during cyclic stretching tests. (**e**) Optical images of the devices at 0, 10%, 20%, and 30% strain. (**f**) Relative resistance change (ΔR/R_0_) of the devices during 1000 stretching cycles at 30% strain, with the inset showing an optical image of the devices. (**g**) Enlarged view of the cyclic stretching response.

Flexible LMI-PVP-PDMS conductive devices were fabricated using the laser-activated LMI-PVP patterns as the core conductive layer, followed by electrode connection, adhesive coating, and tape encapsulation. Electrical stability under storage and repeated mechanical deformation was evaluated to assess the reliability of the printed conductive patterns. Overall, the as-fabricated devices exhibited stable electrical performance during storage, bending, and moderate tensile deformation. As shown in Figure 5a, printed patterns exhibited negligible resistance drift of less than 1% after 14 days of static storage at 25 °C and 60% relative humidity, demonstrating excellent electrical stability under the tested conditions. Upon bending from 0° to 180° around a 1 cm diameter cylinder, the device exhibits a fully reversible resistance response, with ΔR/R_0_ remaining below 1% after 3000 cycles (Figure 5b,c). Under repeated moderate tensile deformation at 30% strain, the device maintained stable electrical behavior, with a resistance drift of only about 3% after 1000 cycles (Figure 5f,g). Notably, Ref. [32] evaluated cyclic resistance under 100% tensile strain with a different substrate and device configuration, whereas this study focuses on PDMS-supported printed conductive patterns. Given that the reliable deformation range is governed by the conductive layer, PDMS interface, and encapsulation configuration, 30% strain was selected to evaluate cyclic electrical stability under moderate tensile deformation rather than to benchmark extreme stretchability. These results confirm reliable electrical stability under storage, bending, and moderate tensile deformation, attributed to the continuous conductive network and strong interfacial adhesion between the laser-activated layer and the PDMS substrate.

The stable electrical response under deformation stems from the synergistic effects of network continuity, interfacial adhesion, and structural protection. First, the dense, continuous LM network formed via multi-solvent regulation provides redundant conductive pathways. This allows electrical transport to be maintained, even when local microcracks are generated during repeated deformation. Second, PVP enhances LM and PDMS interfacial adhesion, effectively suppressing delamination and interfacial sliding. Third, the PDMS encapsulation layer shields against environmental exposure and oxidation. Consequently, the device sustains stable electrical performance under repeated bending and moderate stretching. Stable multimeter readings (Figure S6) confirm that continuous conductive pathways are preserved in flat, bent, and moderately stretched states.

**Figure 6:**
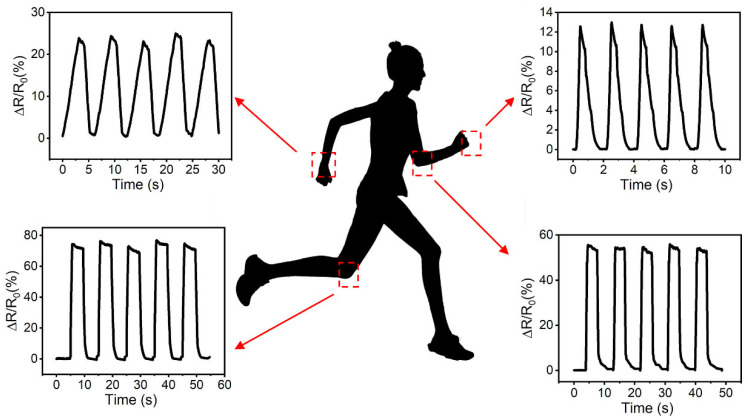
Proof-of-concept demonstration of motion-induced resistance responses using an LMI-PVP-PDMS serpentine printed pattern attached to the finger, wrist, elbow, and knee.

As the primary objective of this study is to develop a screen-printable LM ink and processing route for PDMS-based flexible conductive devices, the body-motion test serves only as a preliminary proof-of-concept demonstration. A serpentine pattern (250 μm line width) was designed to accommodate representative body movements and attached to the finger, wrist, elbow, and knee to record motion-induced resistance responses. As shown in Figure 6, the relative resistance change (ΔR/R_0_) exhibited distinguishable responses during joint movement. These results indicate that the printed conductive pattern generates clear resistance signals under representative body motions. However, quantitative sensor metrics, such as gauge factor, linear sensing range, response/recovery time, and device-to-device repeatability, were not the focus of this work and will be addressed in future studies.

## 4. Conclusions

In this study, a PVP-modified liquid metal composite ink was developed for screen printing on low-surface-energy PDMS substrates using an EtOH/1,2-PG/DMF mixed-solvent system. The mixed-solvent strategy enabled coordinated regulation of wettability, rheological behavior, and drying-induced film formation, enhancing printability on PDMS and yielding uniform, continuous conductive patterns. After laser activation, the printed patterns achieved conductivities of 10^5^ S/m while maintaining stable electrical performance under storage and cyclic deformation. These results confirm the cyclic electrical stability of the printed devices under storage, repeated bending, and moderate tensile deformation. A serpentine printed pattern with a 250 µm line width further demonstrated proof-of-concept motion-induced resistance response. Overall, this work provides an effective materials and processing route for screen-printable LM inks on low-surface-energy PDMS substrates for flexible conductive electronics. Comprehensive sensor characterization, including gauge factor, linearity, response/recovery time, and device-to-device repeatability, will be investigated in future work.

## Figures and Tables

**Table 1:** Comparison of Representative Printable Liquid Metal Ink Systems.

Work	Ink System	Processing Method	Activation Method	Conductivity	Main Focus
Cui et al. [11]	LM–MMT composite ink	Direct writing	Self-conductive	~10^6^ S/m	Wettability improvement and self-conductive LM composite ink
Shang et al. [21]	PVP-based LM ink with propylene glycol	Screen-printing	Water-spray sintering	~10^5^ S/m	Green-solvent LM ink and scalable water-spray sintering
Ma et al [22]	Highly concentrated LM ink with confinement gel strategy	Direct writing	Self-conductive	~10^6^ S/m	High LM loading, but high viscosity limits screen-printing suitability
Lin et al. [23]	High-internal-phase- emulsion gel LM ink	Direct ink writing/3D printing	Drying-induced conductivity	Not specified here	Rheology and shape retention of 3D-printable LM gel ink
Chiu et al. [24]	High-solid-content LM ink	Direct writing	Self-conductive	10^5^–10^6^ S/m	High-solid-content ink and multilayer integration rather than mixed-solvent screen printing
This work	PVP-modified LM ink with EtOH/1,2-PG/DMF	Screen-printing	Laser activation	~10^5^ S/m	Mixed-solvent regulation of wetting, rheology, drying, and laser activation on PDMS

**Table 2:** Surface tension components of probe liquids and their contact angles on PDMS substrates.

Probe Liquid	Total Surface Tension (mN/m)	Dispersive Component (mN/m)	Polar Component (mN/m)	Contact Angle on PDMS (°)
Deionized water	72.8	21.8	51.0	109°
α-Bromonaphthalene	44.4	44.4	0.0	63°

## Data Availability

The original contributions presented in this study are included in the article/Supplementary Material. Further inquiries can be directed to the corresponding author.

## Supplementary Materials

The following supporting information can be downloaded at: https://www.mdpi.com/article/10.3390/s26113279/s1, Figure S1: Sedimentation and phase separation changes in samples from the PVP group and the blank group at different storage times; Figure S2: SEM images of LMI-PVP-E50 at different storage times; Figure S3: Optical microscopy images and corresponding particle size distributions of samples with different LM:PVP mass ratios; Figure S4: Optical microscopy images of LMI-PVP-E50 before and after activation; Figure S5: SEM images and corresponding elemental mapping images of LMI-PVP-E50; Figure S6: Photographs of sensors with different line widths and an LED lighting demonstration.

## References

[1] Chen S., Wang H.-Z., Zhao R.-Q., Rao W., Liu J. (2020). Liquid Metal Composites. Matter.

[2] Zhao Z., Fu H., Tang R., Zhang B., Chen Y., Jiang J. (2023). Failure Mechanisms in Flexible Electronics. Int. J. Smart Nano Mater..

[3] Dickey M.D. (2017). Stretchable and Soft Electronics Using Liquid Metals. Adv. Mater..

[4] Xu B., Ye F., Chang G., Li R. (2020). A Simple and Cost-Effective Method for Producing Stable Surfactant-Coated EGaIn Liquid Metal Nanodroplets. Materials.

[5] Wang S.L., Xu X., Han Z., Li H., Wang Q., Yao B. (2022). Highly Stretchable Liquid-Metal Based Strain Devices with High Sensitivity for Human Activity Monitoring. Mater. Lett..

[6] Liu Y., Yu Z., Gao X., Zhang H., Jiang C., Chen Z., Shang J., Peng L., Li R.-W. (2025). A Recyclable, Ecofriendly, and Biofriendly Biomass-Based Elastomer via the Nanoactivation Effect of Liquid Metal for Electronic Skin. ACS Appl. Mater. Interfaces.

[7] Xu B., Peng W., He J., Zhang Y., Song X., Li J., Zhang Z., Luo Y., Meng X., Cai C. (2024). Liquid Metal-Based Triboelectric Nanogenerators for Energy Harvesting and Emerging Applications. Nano Energy.

[8] Yan Y., Ma J., Ban Y., Li W., Zhang Q., Huang X. (2025). Techniques to Develop Large-Area Multilayer Flexible Antennas Based on Liquid Metals. Appl. Mater. Today.

[9] Pak S., Bartlett M.D., Markvicka E.J. (2024). Direct Ink Write 3D Printing of Fully Dense and Functionally Graded Liquid Metal Elastomer Foams. Adv. Funct. Mater..

[10] Pei D., Dai Y., Dai F., Liang K., Zhao Y., Li Y. (2026). Self-Assembled Aqueous Liquid Metal Inks for Stretchable Conductors and Circuits. npj Flex. Electron..

[11] Cui Z., Zhang Y., Chen S., Wen X., Zhao Y., Ma Y., Yan Q., Wu Z., He Y., Wang G. (2025). A Printable Liquid Metal–Montmorillonite Ink for High-Resolution Stretchable Bioelectronics. J. Mater. Chem. C.

[12] Chen W., Tang Q., Zhong W., Lai M., Shi S., Tan J., Luo Z., Liu X., Ye Z., He R. (2025). Directly Printable and Adhesive Liquid Metal Ink for Wearable Devices. Adv. Funct. Mater..

[13] Liu S., Xu Z., Li G., Li Z., Ye Z., Xu Z., Chen W., Jin D., Ma X. (2023). Ultrasonic-Enabled Nondestructive and Substrate-Independent Liquid Metal Ink Sintering. Adv. Sci..

[14] Thiyagarajan K., Rahul S.G., Adarsh T., Arya P.U., Babu T.A., Rajini G.K., Maji D. (2025). Screen-Printed Flexible Electronic Devices: A Review. Sens. Actuators A Phys..

[15] Xu C., Willenbacher N. (2018). How Rheological Properties Affect Fine-Line Screen Printing of Pastes: A Combined Rheological and High-Speed Video Imaging Study. J. Coat. Technol. Res..

[16] Liu H., Peng Y., Sun J., Zhang Y., Long J., Gu Y., Park S., Liu T., Dong J., Huang Y. (2025). Proanthocyanidin-Enhanced Wettability and Adhesion in Liquid Metal Inks for Multi-Substrate Patterning in Soft Electronics. J. Mater. Chem. A.

[17] Naik K., Saquib M., Shetty S., Holla S.R., Nayak R., Selvakumar M., Rout C.S. (2025). Recent Advances in Screen Printable Microsupercapacitors for Emerging Printed Electronics. J. Energy Storage.

[18] Islam N., Das M., Johan B.A., Shah S.S., Alzahrani A.S., Aziz M.A. (2025). Multifunctional Screen-Printed Conductive Inks: Design Principles, Performance Challenges, and Application Horizons. ACS Appl. Electron. Mater..

[19] Pyeon J., Lee H., Choe W., Park S., Kim H. (2025). Versatile Liquid Metal Composite Inks for Printable, Durable, and Ultra-Stretchable Electronics. Small.

[20] Wang L., Liu J. (2019). Advances in the Development of Liquid Metal-Based Printed Electronic Inks. Front. Mater..

[21] Shang J., Mohammadi M., Strandberg J., Petsagkourakis I., Åhlin J., Hagel O., Yi Y., Herlogsson L., Tybrandt K. (2025). Fully Screen Printed Stretchable Liquid Metal Multilayer Circuits Using Green Solvents and Scalable Water-Spray Sintering. npj Flex. Electron..

[22] Ma R., Jia L., Guo Z., Lin J., Liu M., Wang Z., Song G., Yan D., Li Z. (2025). A General Finite-Gel Strategy for Highly Concentrated Liquid Metal Inks. Nat. Commun..

[23] Lin Z., Qiu X., Cai Z., Li J., Zhao Y., Lin X., Zhang J., Hu X., Bai H. (2024). High Internal Phase Emulsions Gel Ink for Direct-Ink-Writing 3D Printing of Liquid Metal. Nat. Commun..

[24] Chiu Y.-C., Kuo F.-C., Lin Y.-H., Liao Y.-C. (2025). High Solid Content Liquid Metal Ink for Flexible Printed Circuits: Formulation, Stability, and Multi-Layer Integration. Adv. Mater. Technol..

[25] Owens D.K., Wendt R.C. (1969). Estimation of the Surface Free Energy of Polymers. J. Appl. Polym. Sci..

[26] Efimenko K., Wallace W.E., Genzer J. (2002). Surface Modification of Sylgard-184 Poly (Dimethyl Siloxane) Networks by Ultraviolet and Ultraviolet/Ozone Treatment. J. Colloid Interface Sci..

[27] Saikiran P., Purnima D., Mukherjee R., Bhandaru N. (2025). Synergistic Influence of Substrate Wettability and Topography on Surface Phase Separation in PS/PMMA Blend Thin Films. Soft Matter.

[28] Zhang M., Li G., Huang L., Ran P., Huang J., Yu M., Yuqian H., Guo J., Liu Z., Ma X. (2021). Versatile Fabrication of Liquid Metal Nano-Ink Based Flexible Electronic Devices. Appl. Mater. Today.

[29] Larson R.G. (1999). The Structure and Rheology of Complex Fluids.

[30] Deegan R.D., Bakajin O., Dupont T.F., Huber G., Nagel S.R., Witten T.A. (1997). Capillary Flow as the Cause of Ring Stains from Dried Liquid Drops. Nature.

[31] Okuzono T., Ozawa K., Doi M. (2006). Simple Model of Skin Formation Caused by Solvent Evaporation in Polymer Solutions. Phys. Rev. Lett..

[32] Nishikawa T., Yamane H., Matsuhisa N., Miki N. (2023). Stretchable Strain Sensor with Small but Sufficient Adhesion to Skin. Sensors.

