# Supplementary material for: Development of a Screen-Printable Liquid Metal Ink on PDMS Substrates Toward Flexible Conductive Electronics
Source: Sensors (Basel). 2026 May 22;26(11):3279. doi: 10.3390/s26113279 (PMC13259296; doi:10.3390/s26113279)
Supplement: Supplementary file 1 [file sensors-26-03279-s001.zip › sensors-4293317-supplementary.pdf]

Supplementary Material

Development of a Screen-Printable Liquid Metal Ink for PDMS Substrates toward Flexible Electronics

Mengwen Guo[1], Shengming Jin[1,2], Sanhu Liu[3], Fang Wang[3]
1 School of Minerals Processing and Bioengineering, Central South University, Changsha 410083, China
2 Key Laboratory for Mineral Materials and Application of Hunan Province, Central South University, Changsha 410083, China

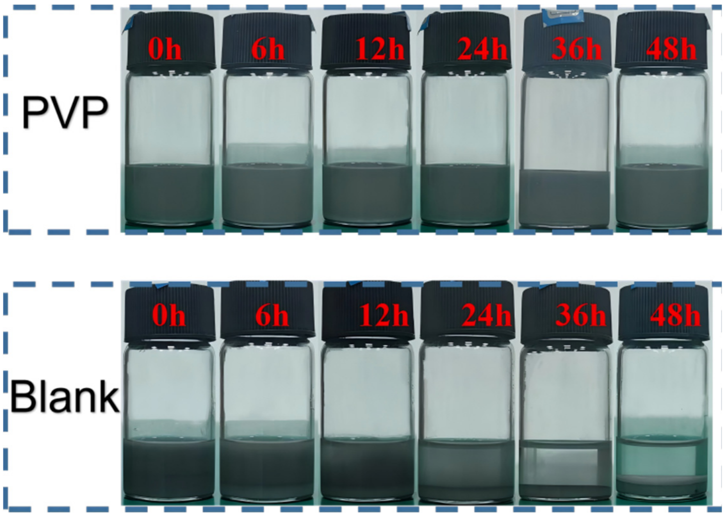

Figure. S1. Sedimentation and phase separation changes in samples from the PVP group and the blank group at different standing times. The upper row shows the PVP group, and the lower row shows the blank group. The observation time points were 0, 6, 12, 24, 36, and 48 h.

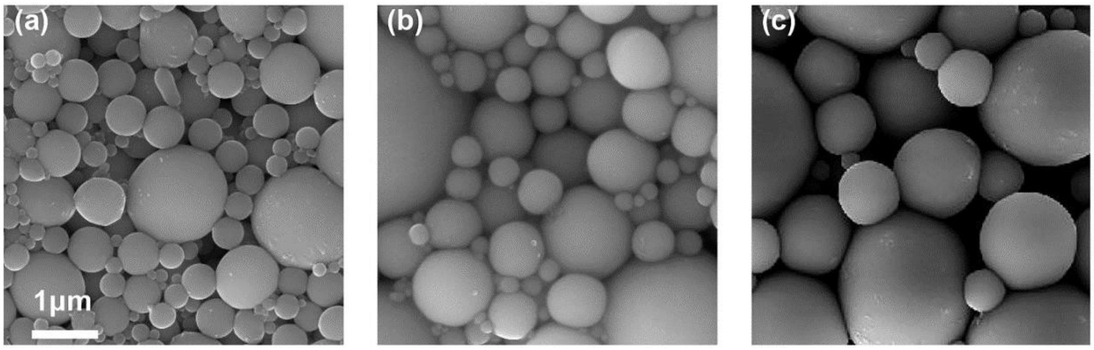

Figure. S2. SEM images of LMI-PVP-E50 at different storage times (0, 2, and 14 d). (a) 0 d; (b) 2 d; (c) 14 d. Scale bar = 1 μm.

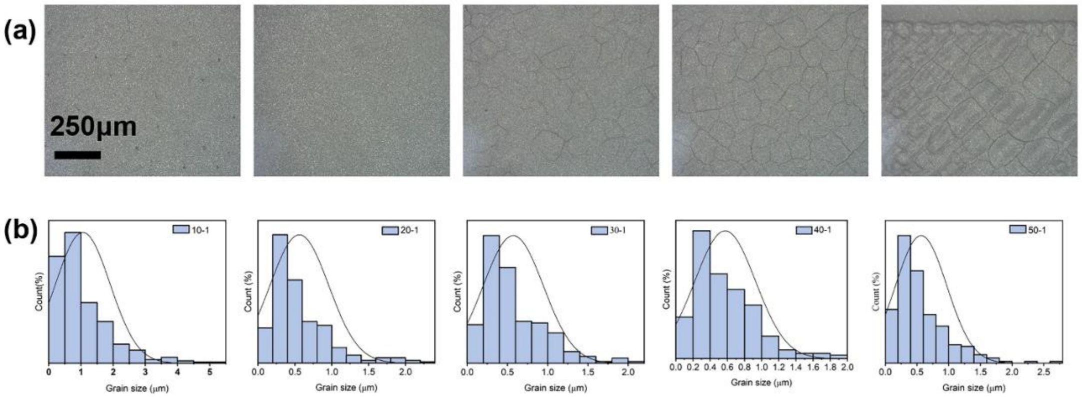

Figure. S3. Optical microscopy images and corresponding particle size distributions of samples with different LM:PVP mass ratios. (a) Optical microscopy images of samples with LM:PVP mass ratios of 10:1, 20:1, 30:1, 40:1, and 50:1; (b) corresponding particle size distribution histograms. Scale bar = 250 μm.

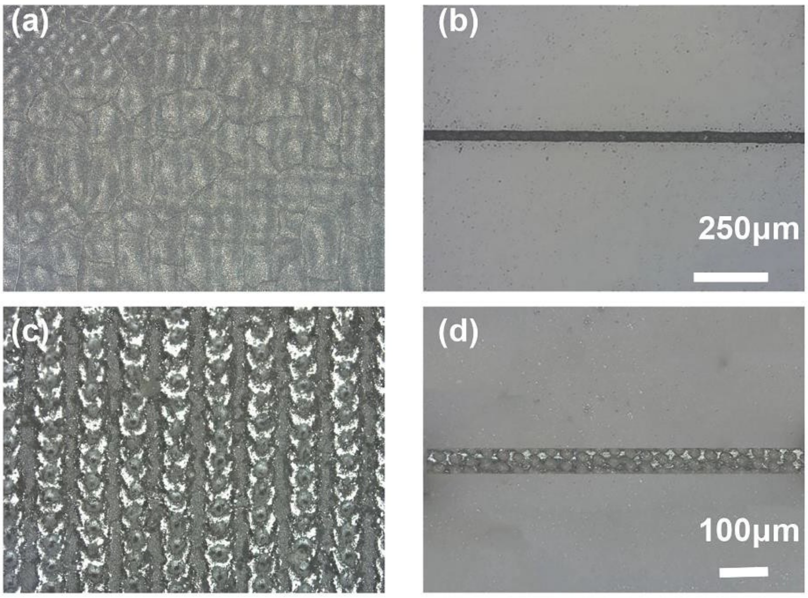

Figure. S4. Optical microscopy images of LMI-PVP-50 before and after activation. (a, c) Samples with a line width of 5000 μm before and after activation, respectively; (b, d) samples with a line width of 50 μm before and after activation, respectively. Scale bars: (a,b) 250 μm; (c,d) 100 μm.

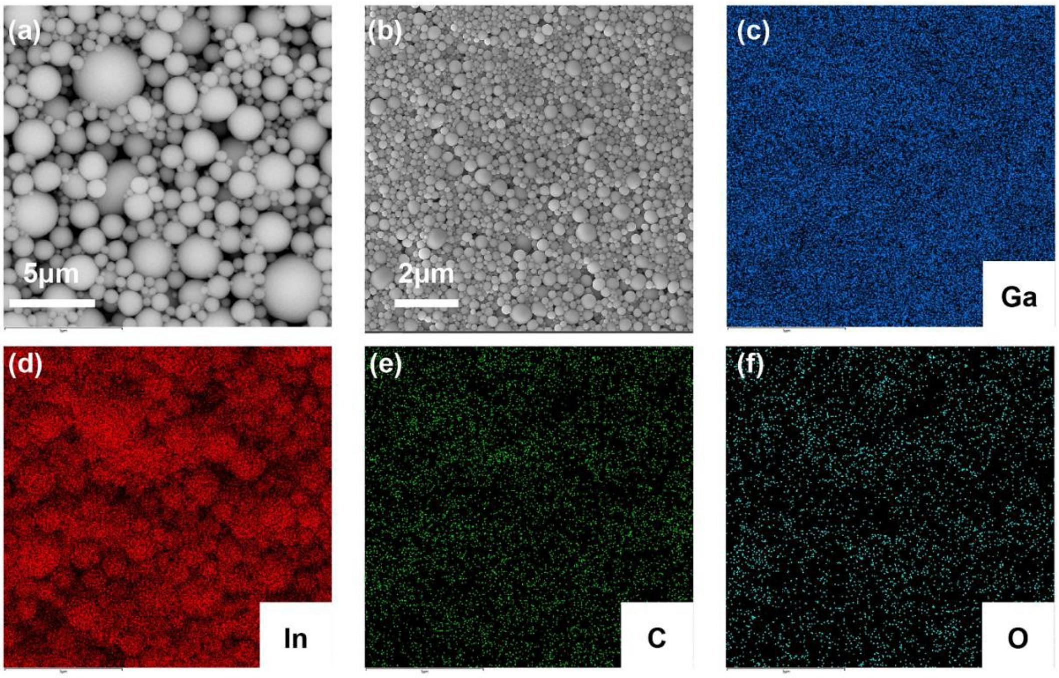

Figure. S5. SEM images and corresponding elemental mapping images of LMI-PVP-E50. (a, b) SEM images of the sample; (c–f) EDS elemental mapping images of Ga, In, C, and O, respectively. Scale bars: (a) 5 μm and (b) 2 μm.

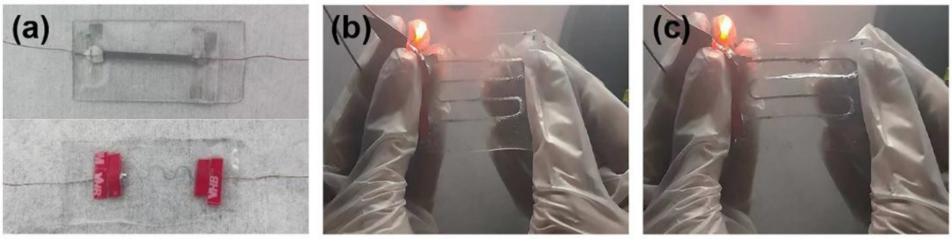

Figure. S6. Photographs of sensors with different line widths and LED lighting demonstration. (a) Photographs of serpentine sensors with line widths of 5000 μm and 250 μm, shown in the upper and lower panels, respectively; (b, c) photographs of the 5000 μm serpentine sensor connected to the circuit before stretching (b) and after stretching (c).
